# Prevalence and Correlates of HIV-Associated Neurocognitive Disorders (HAND) in Northwestern Nigeria

**DOI:** 10.1155/2015/486960

**Published:** 2015-08-09

**Authors:** Ahmad M. Yakasai, Mustafa I. Gudaji, Hamza Muhammad, Aliyu Ibrahim, Lukman F. Owolabi, Daiyabu A. Ibrahim, Musa Babashani, Muhammad S. Mijinyawa, Musa M. Borodo, Abayomi S. Ogun, Abdulrazaq G. Habib

**Affiliations:** ^1^Infectious and Tropical Diseases Unit, Public Health and Diagnostic Institute, College of Medical Sciences, Northwest University, PMB 3220, Kano, Nigeria; ^2^Department of Psychiatry, Aminu Kano Teaching Hospital and Bayero University Kano, PMB 3452, Kano, Nigeria; ^3^Infectious and Tropical Diseases Unit, Department of Medicine, Aminu Kano Teaching Hospital and Bayero University Kano, PMB 3452, Kano, Nigeria; ^4^Neurology Unit, Department of Medicine, Aminu Kano Teaching Hospital and Bayero University Kano, PMB 3452, Kano, Nigeria; ^5^Department of Medicine, Aminu Kano Teaching Hospital and Bayero University Kano, PMB 3452, Kano, Nigeria; ^6^Neurology Unit, Department of Medicine, Obafemi Awolowo Teaching Hospital, Ogun State University, PMB 2002, Sagamu, Ogun State, Nigeria

## Abstract

HIV-associated Neurocognitive Disorders (HAND) are common among HIV-positive individuals. This study explored the prevalence and correlates of HAND in Nigeria. 80 HIV-positive and 40 HIV-negative adults selected from Aminu Kano Teaching Hospital (AKTH) received comprehensive evaluations. A multidomain neuropsychological test (MDNPT) battery assessing 7 domains was administered to the participants and their performance was combined with measures of functional status to classify impairments into various grades of HAND. Univariate and multivariate analyses were performed to identify correlates of symptomatic HAND. Among the HIV-positive individuals, 50% were highly active antiretroviral therapy-experienced (HAART+) and 50% were highly active antiretroviral therapy naive (HAART−). Symptomatic HAND was found among 40% of the HAART− individuals and 30% of the HAART+ individuals. Respective prevalence of HIV-associated dementia (HAD) was 23% and 5%, respectively (*p* = 0.0002). In a binary logistic regression model, only fewer years of education independently predicted symptomatic HAND [Odds Ratio (OR) = 1.2, 95% confidence interval (CI) = 1.04–1.44, *p* = 0.016]. The prevalence of HAND in Nigeria is high with HAD being commoner among HAART− patients. Provision of HAART and strict monitoring of patients at risk of HAND are needed to scale down the burden of the disease.

## 1. Introduction

Based on the weaknesses of the 1991 American Academy of Neurology (AAN) classification scheme observed over time, the AAN working group in 2007 convened in Frascati, Italy, and redefined the neurocognitive manifestations of HIV-1 infection [1]. The three grades of HAND identified in the updated nosology in order of increasing severity were asymptomatic neurocognitive impairment (ANI), mild neurocognitive disorder (MND) and HIV-associated dementia (HAD). Although this classification is mainly for research purposes, it has both clinical relevance and epidemiological relevance [1]. HAD is an Acquired Immune Deficiency Syndrome (AIDS) defining illness associated with poor clinical outcome, low CD4 count, high viral load, low haemoglobin, and poor adherence to ART [2–7]. Few studies in Sub-Saharan Africa (SSA) have reported these factors in addition to other parameters as correlates of HIV-related NCI [2–7].

In Western countries, the incidence of HAD was fairly stable across monotherapy era in early 1990s through dual therapy era in mid 1990s [8]. From 1996, the introduction of HAART has led to a sharp reduction in the incidence of HIV dementia by about 50% [8]. Over the same period the mean/median CD4 count of individuals with HIV dementia was rising [8]. In the Multicenter AIDS Cohort Study (MACS) the mean CD4 count of newly diagnosed HIV dementia cases rose from 151 in 1992 to 518 in 2001 [8].

As reported in a meta-analysis, more than 8.1 million individuals in SSA may be affected by symptomatic HAND [9]. However, the neuroepidemiology of this condition still remained understudied in SSA and other parts of the world. Most of the studies undertaken so far using standard tools and criteria emanated from the industrialized world and very few came from SSA, the region bearing the highest burden of HIV infection. A wide gap exists between the industrialized world and SSA in terms of antiretroviral therapy (ART) utilization, CD4 threshold for commencement of ART, burden of HIV infection, availability of standard neuropsychological evaluation tools, and demographically adjusted normative data [10]. Thus it may be inappropriate to extrapolate figures from industrialized world to resource-constrained countries of SSA given the obvious sociodemographic and epidemiologic differences between the two regions.

Nigeria bears the second highest burden of HIV/AIDS in the world providing a large pool of patients for neuroAIDS study [10]. However, the prevalence and correlates of HAND have not been established in Nigeria using the standard multidomain neuropsychological testing. The reported prevalence of NCI among HIV infected patients in Nigeria varied widely ranging from 28.8% on the international HIV dementia scale (IHDS) to 66.7% on the community screening instrument for dementia (CSI-D) [11, 12], while the prevalence of HAND using brief tools was reported to be 21.5% [7]. Determining the actual prevalence estimates of HAND in Nigeria could positively impact scale-up of ART, resource allocation, budgeting, and planning for HIV/AIDS. In the same vein, appreciating correlates of HAND could be instrumental for early detection of at risk patients for early intervention.

## 2. Methodology

This cross-sectional study was conducted at Aminu Kano Teaching Hospital (AKTH) in Northwestern Nigeria. Eighty HIV-positive individuals and 40 HIV-negative individuals who were matched in terms of gender, age, and level of education were recruited. The later provided the comparison group for rating and classification of impairment due to lack of demographically adjusted published normative data in Nigeria. The HIV-positive individuals were selected from the HIV/AIDS clinic, whereas the HIV-negative control individuals were selected from the HIV Voluntary Counseling and Testing (VCT) unit and blood donor clinic. Before commencement of the study, ethical approval was obtained from the research ethics committee of AKTH.

### 2.1. Inclusion and Exclusion Criteria

Individuals who met the following inclusion criteria were recruited for the study: (1) at least 18 years of age, (2) should not meet the criteria for the diagnosis of current substance abuse and/or dependence, for example, alcohol and stimulants, (3) should have no history of psychiatric diseases such as Schizophrenia, severe depression, posttraumatic stress disorder, and learning disability, (4) not diagnosed to have Central Nervous System (CNS) opportunistic infections like Toxoplasmosis and Meningitis, (5) should be free of chronic medical conditions such as systemic hypertension, diabetes mellitus, stroke, Chronic Kidney Disease (CKD), and Chronic Liver Disease (CLD), and (6) should provide written informed consent.

Participants who satisfied any of the following criteria were excluded from the study: (1) currently abusing or dependent on substances including alcohol, (2) positive for Hepatitis C virus antibody, (3) positive serum cryptococcal antigen test, (4) inability to understand commonly used English words, and (5) failed to meet any of the inclusion criteria or did not consent to participate.

### 2.2. Neuromedical Evaluation

Study participants received detailed evaluation in terms of medical history, drug history, and social history via interview by clinicians. Neuromedical screening handout, substance-use handout, substance-use history handout, academic skills handout, and behavioral handout were filled by study individuals. The academic skills questionnaire contained questions about reading, writing, simple addition/subtraction, and understanding simple written instructions on packaged items. The behavioral handout that was also administered to the study individuals assessed confounds alcohol, drugs, head injury, language, education, comorbidities, handedness, ethnicity, effort, employment, and transportation. Other items also briefly assessed in the behavioral handout include gait disturbances, use of hands, speech, demeanor, affect, rapport, cooperation, distractibility, attention/concentration, frustration tolerance, understanding instruction, and auditory/visual functions. Subsequently detailed physical examination was performed to assess neurological function and ensure that recruited individuals were free of confounders like focal neurologic deficit, stroke, and opportunistic infections of the CNS.

### 2.3. Neuropsychological Assessment Tools

Comprehensive multidomain neuropsychological tests (MDNPT) battery was administered to all the study participants. These tests covered the seven ability domains typically affected by HIV-related neurocognitive dysfunction and comprised the following: memory, Hopkins verbal learning test-revised (HVLT-R) delayed recall and trial recognition; speed of information processing (SIP), Wechsler adult intelligence scale III (WAIS-III) symbol search; motor, grooved pegboard dominant hand (DH) and nondominant hand (NDH); abstraction/executive function, color trails 2; learning, HVLT-R immediate recall; attention/working memory, Wechsler memory scale III (WMS-III) spatial span; verbal fluency, controlled oral word association test (COWAT) [13]. Screening for effort was done with Hiscock Digit Memory Test (HDMT). These tests were extracted from the international neurobehavioral tests battery used by the HIV Neurobehavioral Research Center (HNRC) and have been found to be sensitive to HAND in Nigeria [11, 14]. In addition, selection of these tests was further guided by their simplicity and most required no knowledge of English language except HVLT-R and COWAT tests which need understanding of some English words. Among the authors, AGH had training on administration of these neuropsychological tests at HNRC and was involved in a previous study in Nigeria using similar tests [11]. He in turn trained and supervised AMY and MIG who administered the battery tests in this study.

### 2.4. Assessment of Functional Status and Depression

Assessment of functional status was done using the following questionnaires: Personal Assessment of Own Functioning Inventory (PAOFI), Karnofsky performance scale, and Instrumental Activities of Daily Living (IADL). PAOFI assessed memory, language/communication, employment, use of hand, sensory/perceptual function, and higher level cognitive and intellectual functions. ADL assessed house-keeping, financial management, shopping, cooking, planning social activities, understanding reading materials, transportation, and using telephone. Other items also assessed by ADL include home repairs, bathing, dressing, laundry, taking medication, child care, and work. Depression was assessed with Beck Depression Inventory (BDI-II). All these instruments have been validated in Nigeria in collaboration with HNRC researchers [11, 14].

### 2.5. Classification of Neurocognitive Impairments

The 2007 modified American Academy of Neurology (AAN) criteria (also known as Frascati criteria) were used to classify neurocognitive impairments. Here individuals were classified in order of increasing severity as ANI, MND, and HAD. ANI refers to abnormal performance ≥1 standard deviation (SD) below the mean of demographically matched HIV-negative comparator group in two or more neurocognitive domains without impairment in functional status. MND refers to mild cognitive dysfunction ≥1 SD below the mean of demographically matched HIV-negative comparator group in at least two neurocognitive domains in presence of mild impairment in functional status. Individuals with profound cognitive dysfunction ≥2 SD below the mean of demographically matched HIV-negative comparator group in two or more neurocognitive domains in presence of profound impairment in functional status were classified as having HAD [1]. A score of ≥17 on BDI was considered significant. Functional impairment on PAOFI was defined as ≥2 cognitive complaints, whereas impairment on IADL was defined as decline in ≥2 areas [1]. These measures were combined to define functional decline as outlined in the updated 2007 nosology for HAND [1].

### 2.6. Assessment of Adherence to Antiretrovirals (ARV)

Adherence to ARV was determined through self-report and defined as “always adherent” or “not always adherent”.

### 2.7. Laboratory Assessment

All study participants had rapid HIV test, hepatitis B surface antigen test, hepatitis C antibody test, and Venereal Disease Research Laboratory (VDRL) test. For HIV-positive individuals other additional tests performed include serum cryptococcal antigen, CD4 cell count, and viral load assay (available for only 44 individuals).

## 3. Statistical Analysis

Neuropsychological tests scores of HIV-positive and HIV-negative individuals were compared using Student's *t*-test. Across the grades of HAND, categorical variables were compared using Chi-square test, whereas continuous variables were compared using one-way analysis of variance (ANOVA) and Kruskal-Wallis test where appropriate. Apart from age, all the other continuous variables were not normally distributed and, even after logarithmic transformations, current CD4 count, VL, Hb, and Karnofsky score were still significantly skewed; hence, we used Kruskal-Wallis test to analyze them. Variables that showed significant association with HAND in the univariate analysis were included in the multivariate analysis using a binary logistic regression model to determine independent predictors of symptomatic HAND. Here, individuals with symptomatic HAND were compared with neurocognitively unimpaired HIV-positive individuals. Data analysis was performed using Statistical Package for Social Sciences (SPSS) version 18 and statistical significance was set at *p* ≤ 0.05.

## 4. Results

### 4.1. Characteristics of Study Participants

Out of the 8 HIV-positive individuals excluded from the study, 6 were positive for serum cryptococcal antigen test, one was reactive for hepatitis C virus antibody, and one scored below the 90% cut-off score on HDMT. Sixteen (20%) of the HIV-positive individuals had history of alcohol ingestion remotely in the past but none of them met criteria for current alcohol abuse and or dependence. All cases and controls were at least 18 years of age and well matched with regard to age, sex, and years of education (see Table 1). Overall, 80 HIV-positive and 40 HIV-negative individuals were evaluated and their primary languages were Hausa (81%), Yoruba (6%), Igbo (8%), and others (5%). Among the 80 HIV-positive individuals, 38 (48%) have reached AIDS stage, 40 (50%) were HAART+, and 40 (50%) were HAART−. The 40 HAART+ HIV-positive individuals have received HAART for a mean of 4 (2.17) years. Twenty six (65%) of them were on Nevirapine containing regimen and 14 (35%) were on Zidovudine containing regimen. The median CNS penetration effectiveness (CPE) of the antiretroviral agents was 2.0 (1.0–2.5), with 27 (68%) having CPE rank ≥2.0. Viral load assay was available for only 44 HIV-positive individuals (40 HAART+ and 4 HAART−). Twenty nine (73%) of the HIV-positive HAART+ individuals had undetectable viral load (<400 copies/mL), while all the 4 HIV-positive HAART− individuals had detectable viral load. The mean CD4 counts for HAART+ and HAART− individuals were 367 and 261 cells/mL, respectively (*p* = 0.045).

### 4.2. Neurocognitive Performance of Study Participants

The HIV-positive individuals performed worse than the HIV-negative individuals in all the domains as shown in Table 2. Statistically significant difference was found in all the domains except SIP (WMS-III spatial span) and executive function (color trails 2).

### 4.3. Prevalence and Correlates of HAND

**Figure 1:**
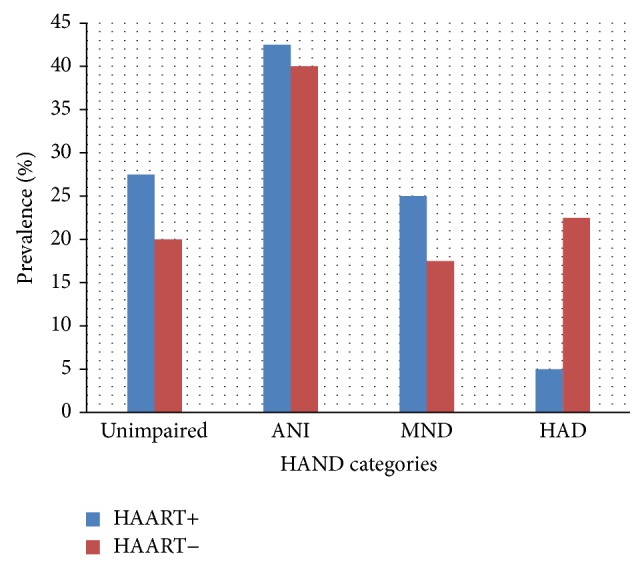
Prevalence of HAND categories among the HIV-positive individuals as defined by Frascati criteria.

Figure 1 shows the prevalence of various grades of HAND. Among the 40 HIV-positive HAART− individuals, symptomatic HAND was found among 40% [MND = 9 (22.5%) and HAD = 7 (17.5%)], 16 (40%) had ANI, and 8 (20%) were neurocognitively unimpaired. For the HIV-positive HAART+ individuals, symptomatic HAND was found among 30% [MND = 10 (25%) and HAD = 2 (5%)], 17 (42.5%) had ANI, and 11 (27.5%) were neurocognitively unimpaired. For the HIV-negative comparison group the prevalence of symptomatic neurocognitive impairment was 18% (mild form = 18% and severe form = 0%). ANI was found in 23% of the HIV-negative individuals.

**Figure 2:**
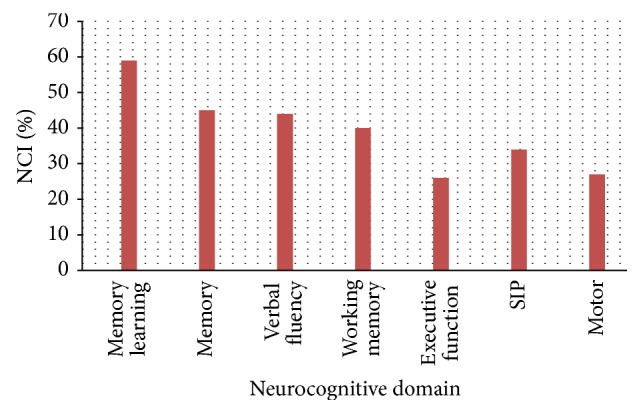
Percentage of the HIV-positive individuals with NCI by neurocognitive domain. NCI was defined as a score ≥2 SD below the mean of the HIV-positive individuals in the neurocognitive domain.

Domain-specific NCI defined as a score ≥2 standard deviations (SD) below the mean of the HIV-negative individuals was highest in verbal learning domain (59%). This was followed by memory (45%), verbal fluency (44%), working memory (40%), SIP (34%), motor (27%), and executive function (26%) domains (see Figure 2).

HIV-positive individuals with poor adherence to HAART had significantly lower Karnofsky score (*p* = 0.001), lower Hb (*p* < 0.0001), lower CD4 count (*p* = 0.003), higher viral load (*p* = 0.001), and poor performance in memory recognition domain (*p* = 0.027). Univariate analysis revealed that years of education, Karnofsky performance score, age, Hb, and depression score significantly differ across the grades of HAND (see Table 3). Individuals with HAD were characterized by fewer years of education, lower CD4 count, lower Karnofsky performance score, lower Hb, and higher BDI score. In a binary logistic regression model comparing HIV-positive unimpaired individuals with those having symptomatic HAND, fewer years of education independently predicted symptomatic HAND (see Table 4).

## 5. Discussion

By utilizing a comprehensive battery sensitive to the subcortical HIV-related neurocognitive deficits, this study found higher prevalence of HAD among HAART+ individuals than among HAART− individuals (23% and 5%, resp., *p* = 0.0002). Majority of the HAART+ individuals in this study had undetectable viral load with significantly higher CD4 count than the HAART− individuals (*p* = 0.045). It is not surprising, therefore, to obtain higher prevalence of HAD among HAART− as compared to HAART+ individuals. The prevalence of symptomatic HAND in this study ranged from 30% to 40% depending on HAART utilization and is higher than the 11.9% reported from a study conducted in a city close to and culturally similar to the city where our study was performed. In that study, brief neuropsychological tests, namely, IHDS, stick design test, and word recall test were administered to a group of individuals comprising 77.8% female with a mean age of 37.2 years [7]. The IHDS is a screening tool used to detect patients at risk of developing HAND who might benefit from further extensive testing with the MDNPT battery [15]. The second test utilized in that study, the stick design test, is a measure of praxis which is predominantly a cortical function. Neuropsychological performance on this test is influenced by age, gender, and education and it has been shown that this test is most suitable in older people of at least 65 years of age [16]. It is not unexpected, therefore, to report lower prevalence of HAND using a screening battery not sensitive to the typical subcortical deficits exhibited by HIV infected individuals. From another related study in Nigeria where ideal neuropsychological testing was utilized, the frequency of HIV-related NCI was 31% among individuals with early stage HIV infection [14]. Similarly, with comprehensive neuropsychological testing in Uganda and South Africa, the prevalence of HAD was found to be 31% and 25%, respectively [3, 4].

Our finding of lower prevalence of HAD in relation to ANI and MND in this study is in keeping with the trend in the HAART era where less severe grades of HAND are more common than the severest grade [17]. In the HAART era, less severe forms of neurocognitive dysfunction are prevalent and more readily picked by the updated nosology in which ANI was recognized as an entity [1]. However, this has generated a lot of controversies and there are concerns about the possibility of exaggeration of impairment and misclassification of patients [18]. Notwithstanding, this form of NCI has clinical relevance as shown in a recent study in which HIV-positive individuals with ANI were longitudinally studied and found to have up to 2–6-fold increased risk of faster progression to symptomatic grades of HAND [19]. In addition, ANI also accelerates decline in functioning measured by self-report or performance-based methods and the condition therefore warrants recognition as a clinically relevant entity [19]. Within the spectrum of HAND, ANI afflicts up to 70% of HIV-positive individuals and could potentially serve as point of strategic intervention to halt progression to symptomatic HAND categories [20].

The transition from pre-HAART to HAART era has created a shift in the neurocognitive profile of HAD. In the pre-HAART era, ADC is a relentless and progressive subcortical disease characterized by impaired motor function, SIP, and verbal fluency. In the HAART− era, however, impairments in memory (learning) and executive function are more common among patients with HAD who may have both cortical and subcortical pathologies [21]. In this study, domain-specific neurocognitive impairment was highest in verbal learning and memory domains and is typical of what is expected in the HAART era. This finding is further supported by the statistically strong association between HIV infection and performance in memory and verbal learning domains (*p* < 0.0001).

The cerebral reserve hypothesis has been used to explain the relationship between educational level and risk of dementia [22]. Low educational attainment is directly associated with lower cognitive reserve thereby increasing the risk of developing dementia [22]. The finding of few years of education as an independent risk factor for cognitive impairment in this study is similar to the report of other studies from South Africa [4], Botswana [6], and Cameroon [2]. In contrast, these studies additionally reported older age to be associated with cognitive impairment. Although lower educational level has been found to be associated with neurocognitive impairment across several studies [2, 4, 6], it could, however, be an erroneous predictor of HAD since individuals with lower education could score lower on neurocognitive tests [22]. Neuropsychological scores on two of the tests requiring knowledge of English (HVLT-R, delayed recall and immediate recall) in this study are similar to the scores obtained in a study from China using similar tool and criteria [23]. However, scores on the test of letter fluency (COWAT) are relatively low and contrast sharply with the finding of Royal III et al. in a study from Nigeria where they reported participants having high score in that test. Perhaps this may be related to the higher level of education of the participants in that study since >39% of them had education after high school [11]. It is imperative to note that, in setting with lower educational level, administration of tests in English language may lead to lower scores especially in tests requiring knowledge of English language [24]. In order to reduce the confounding effect of English language among Nigerians, there is a need to translate the international neurobehavioral battery into the major Nigerian languages.

Poor adherence to HAART in this study is associated with lower Karnofsky score, lower CD4 count, lower Hb, and higher plasma HIV RNA. Comparison in terms of cognitive domains showed no significant difference between the two groups except in the domain of memory (delayed recognition) where nonadherent subjects significantly performed worse than adherent subjects (see Table 5). This finding is corroborated by earlier report that memory impairment is associated with poor adherence [25]. However, in contrast to our finding, the earlier report also showed that poor adherence was associated with executive function and psychomotor slowing. This discrepancy could be attributed to the self-reported measure of adherence employed in the current study, which might have underestimated the level of nonadherence. Electronic monitoring device (medication event monitoring system caps) employed in the other study may be more precise in assessing medication adherence.

Lack of demographically adjusted normative scores of neuropsychological tests has been a major threat to conducting neuroAIDS studies in SSA. Two previously conducted studies in Uganda and South Africa had provided normative data for selected neuropsychological tests. Robertson et al. administered the Auditory Verbal Learning Test (AVLT), symbol digit modalities test, color trails 1 and 2, timed gait, grooved pegboard (dominant and nondominant), and digit span (forward and backward) [26]. Singh et al. on the other hand administered trail making test A and B, digit span (forward and backward), and the IHDS memory subset [27]. In a study conducted in Nigeria using more detailed neuropsychological testing, age and education adjusted scores for comparison were generated from 77 HIV-negative individuals. Because of the small sample size and the fact that only those fluent in English language were selected, the values generated may not be reasonably accurate to serve as population normative data. Therefore, in this study, we generated comparison scores from age and education matched HIV-negative individuals due to lack of appropriate population normative data in Nigeria.

In addition to above limitations, this study has other limitations worthy of note. Firstly, the study was hospital-based and therefore generalizability to the community may be limited. Secondly, neuroimaging was not conducted due to financial reasons. However, CNS opportunistic infections were rigorously assessed by detailed neuromedical history, clinical examination, and serum cryptococcal antigen test. Thirdly, cut-offs used to define functional impairment on PAOFI and IADL were mainly derived from western population and their validity in Africans needs to be confirmed. Despite these limitations, we believe results from this study are reliable because efforts were made at controlling the confounding effect of sociodemographic factors like age, sex, and education. Even though exclusion of subjects with comorbidities further increases the reliability of the results obtained in our study, the final prevalence rates arrived at could have been deflated. It has been well documented in the CNS HIV Antiretroviral Therapy Effects Research (CHARTER) study that patients with higher level of comorbidities tend to have greater prevalence of HAND [20].

In view of the high prevalence of HAND observed in this study, clinicians, healthcare workers, and stakeholders involved in HIV/AIDS care should pay more attention to this condition. HIV-positive individuals with lower educational level are at higher risk of developing HAND, a condition associated with poor ART adherence [28]. Neurocognitive assessment should therefore be offered to all HIV-positive patients at risk of HAND to avoid progression to symptomatic grades of HAND. Even though the MDNPT battery is not readily available in Nigeria, simpler tools that have good psychometric properties for HAND screening like the IHDS and the CSI-D could be used to routinely evaluate patients [11, 12]. Larger studies are needed in Nigeria to further characterize HAND and explore other correlates of neurocognitive function among HIV-positive individuals.

## Figures and Tables

**Table 1:** Characteristics of the study participants.

Characteristics	HIV-positive (*n* = 80)	HIV-negative (*n* = 40)	Chi-squared	*p* value
Age^*∗*^	36.76 (8.97)	34.78 (9.62)	—	0.266
Education years^*∗*^	12.00 (4.27)	12.73 (2.80)	—	0.333
Gender, *n* (%)				
Males	44 (55)	21 (52.5)	0.067	0.796
Females	36 (45)	19 (47.5)		
BDI score (≥17)	19 (24)	4 (10)	15.86	<0.0001
PAOFI score (≥2)	23 (29)	5 (12.5)	21.70	<0.0001
IADL score (≥2)	13 (16)	1 (2.5)	13.42	0.0002

^*∗*^Mean (SD). BDI, Beck Depression Inventory; PAOFI, Personal Assessment of Own Functioning; IADL, Instrumental Activities of Daily Living.

**Table 2:** Neuropsychological tests score of HIV-positive and HIV-negative individuals.

Domain and NP test	HIV-positive (*n* = 80)	HIV-negative (*n* = 40)	*p* value
*SIP *			
WAIS-III symbol search	18.31 (7.45)	19.77 (6.45)	0.292
*Attention/working memory*:			
WMS-III spatial span	10.39 (3.04)	11.78 (2.43)	0.013
*Memory *			
HVLT-R (delayed recall)	6.49 (1.96)	7.90 (1.85)	<0.0001
Trial recognition	21.40 (2.29)	22.35 (1.41)	0.018
*Verbal learning *			
HVLT-R (immediate recall)	18.26 (4.17)	22.33 (4.00)	<0.0001
*Verbal (letter) fluency *			
COWAT-FAS	17.55 (8.92)	22.65 (7.11)	0.002
*Motor *			
Grooved pegboard DH	88.18 (33.99)	75.43 (22.58)	0.034
Grooved pegboard NDH	109.01 (89.85)	88.85 (26.93)	0.028
*Abstraction/executive functioning *			
Color trails 2	251.01 (154.80)	210.25 (80.88)	0.061

Data are mean (SD). COWAT, controlled oral word association test; DH, dominant hand; HVLT-R, Hopkins verbal learning test-revised; NDH, nondominant hand; NP, neuropsychological; SIP, speed of information processing; WAIS-III symbol search, Wechsler adult intelligence scale; WMS-III, Wechsler memory scale.

**Table 3:** Demographic, clinical, and laboratory characteristics of HIV-positive individuals across the HAND categories.

Characteristics	AAN-defined HAND categories, *n* (%)				Statistical value^‡^
	Unimpaired	ANI	MND	HAD	
	19 (24)	33 (41)	17 (21)	11 (14)	
Age, (years)^*∗*^	36.89 (8.32)	36.97 (8.77)	36.35 (10.09)	36.55 (10.10)	*F* = 0.03 *p* = 0.974

Gender, *n* (%)					
Male Female	12 (63) 7 (37)	19 (57.6) 14 (42.4)	8 (47.1) 9 (52.9)	5 (45.5) 6 (54.5)	*X* ^2^ = 1.44 *p* = 0.697

Education, (years)^*∗*^	13.79 (2.57)	12.15 (4.09)	12.29 (3.98)	8.00 (5.44)	*F* = 5.0 *p* = 0.003

AIDS, *n* (%)					
Yes No	7 (37) 12 (63)	16 (48.5) 17 (51.5)	8 (47.1) 9 (52.9)	7 (63.6) 4 (36.4)	*X* ^2^ = 2.0 *p* = 0.57

Karnofsky score^†^	100 (80–100)	90 (50–100)	90 (70–100)	80 (50–80)	*X* ^2^ = 24.5 *p* < 0.0001

CPE score, *n* (%)					
≤2 >2	6 (54.5) 5 (45.5)	10 (58.8) 7 (41.2)	6 (60) 4 (40)	0 (0) 2 (100)	*X* ^2^ = 2.65 *p* = 0.45

Current CD4 count^†^	182 (72–1226)	294 (19–926)	378 (51–861)	143 (14–675)	*X* ^2^ = 9.4 *p* = 0.025

Nadir CD4 count					
<200 ≥200	7 (63.6) 4 (36.4)	12 (70.6) 5 (29.4)	8 (80) 2 (20)	1 (50) 1 (50)	*X* ^2^ = 1.1 *p* = 0.78

Viral load (log_10_)^†^	2.6 (2.6–5.9)	2.6 (2.6–4.9)	5.5 (2.6–5.8)	2.6 (2.6–6.0)	*X* ^2^ = 0.85 *p* = 0.84

Haemoglobin (g/dL)^†^	11.5 (9.2–12.6)	10.9 (7.7–13.8)	10.6 (7.7–13.2)	9.0 (4.4–11.1)	*X* ^2^ = 15.8 *p* = 0.001

BDI score^*∗*^	13.74 (9.60)	8.85 (7.65)	12.59 (8.16)	16.27 (7.98)	*F* = 3.0 *p* = 0.035

ANI, asymptomatic neurocognitive impairment; AAN, American Academy of Neurology; AIDS, Acquired Immune Deficiency Syndrome; BDI, Beck Depression Inventory; CDC, Center for Disease Control and Classification; CPE, CNS penetrating effectiveness; HAD, HIV-associated dementia; IQR, Interquartile Range; MND, mild neurocognitive disorder. ^*∗*^Mean (SD). ^†^Median (IQR). ^‡^Continuous variables analyzed using one-way analysis of variance (ANOVA) and Kruskal-Wallis test as appropriate.

**Table 4:** Multivariate logistic regression model to determine independent predictors of symptomatic HAND among HIV-positive individuals.

Variable	OR	95% CI	*p* value
Few years of education	1.22	1.04–1.44	0.016
Low haemoglobin	1.44	0.85–10.89	0.09
Low Karnofsky score	1.02	0.95–2.21	1.10
Low CD4 count	1.00	0.99–1.00	0.77
Depression	0.98	0.90–1.00	1.06

Model *R* ^2^ = 0.337. CI, confidence interval, OR, Odds Ratio.

**Table 5:** Characteristics of HIV-positive individuals based on adherence to HAART.

Characteristics	HAART adherence status		*p* value
	Adherent (*n* = 30)	Nonadherent (*n* = 10)	
Karnofsky performance score	95.00 (6.82)	83.00 (13.38)	0.001
CPE rank score	2.10 (0.46)	1.80 (0.59)	0.105
CD4 cells/mL	436.30 (262.42)	159.30 (106.95)	0.003
Nadir CD4 cells/mL	152.63 (108.44)	101.20 (104.47)	0.198
Viral load (log_10_ copies/mL)	2.6 (1.6)	5.3 (5.4)	0.001
Haemoglobin	11.15 (1.03)	8.82 (1.86)	<0.0001
Cognitive symptoms (PAOFI)	1.00 (1.46)	1.00 (1.49)	1.00
Activities of daily living (IADL)	0.63 (2.22)	1.50 (1.35)	0.147
Memory (recall) test score	7.20 (1.87)	6.40 (1.79)	0.256
Memory (recognition) test score	22.50 (1.58)	20.97 (22.66)	0.027

Data are mean (SD). CPE, CNS Penetration Effectiveness; HAART, highly active antiretroviral therapy; IADL, Instrumental Activities of Daily Living; PAOFI, Personal Assessment of Own Functioning Inventory.
